# “Social functioning and use of rehabilitation resources in a group of people who experienced a first episode of psychosis and participated in a psychotherapeutic group program versus a control group”

**DOI:** 10.1192/j.eurpsy.2023.443

**Published:** 2023-07-19

**Authors:** A. Oliva Lozano, J. Garde Gonzalez, P. Herrero Ortega, A. Muñoz-Sanjosé, Á. Palao-Tarrero, M. P. Vidal-Villegas, R. Mediavilla, P. Tarín Garrón, J. M. Pastor-Haro, Á. De Diego Gómez-Cornejo, M. F. Bravo-Ortiz, O. B. O. A.-M. Group

**Affiliations:** ^1^Psychiatry, Clinical Psychology and Mental Health, La Paz University Hospital; ^2^Psychiatry, Autonomous University of Madrid (UAM); ^3^ Hospital La Paz Institute for Health Research (IdiPAZ); ^4^ Centro de Investigación Biomédica en Red de Salud Mental (CIBERSAM); ^5^ School of Medicine, Autonomous University of Madrid (UAM); ^6^Psychiatry, Rodriguez Lafora Hospital, Madrid, Spain

## Abstract

**Introduction:**

Psychotic disorders have a huge impact on social functioning, which is the ability to stablish and maintain social activities such as interpersonal relationships and self-care activities of daily living. Research data support that the early intervention in people who have experienced a first episode of psychosis (FEP) -based on a multidisciplinary treatment including both psychopharmacological and psychosocial treatments-, has a relevant role in a favorable evolution. AGES-Mind study is based on manualized psychotherapeutic interventions for people with first-psychosis episodes.

**Objectives:**

To describe the use of rehabilitation resources and social functioning in a group of people with FEP who were included in a psychotherapeutic group program versus a control group, at 12 and 24 months since the beginning of the intervention.

**Methods:**

Longitudinal, analytical, observational, retrospective study on a cohort of 46 patients with first-episode psychosis within the last 5 years. 23 patients received group psychotherapy in the context of the AGES-Mind study and they were compared with 23 control patients who did not receive a group intervention (treatment as usual). Controls were matched by age, gender and time elapsed since the first episode of psychosis with those exposed to the intervention. Sociodemographic data, social functioning (self-care, social activities, social relationships, and behavior) and use of rehabilitation resources outcome variables were assessed.

**Results:**

Significant differences were found regarding participation in social activities in the intervention group versus control group at 24 months. No significant differences were found in other dimensions of social functioning or in the use of rehabilitation resources.

**Image:**

**Figure:**
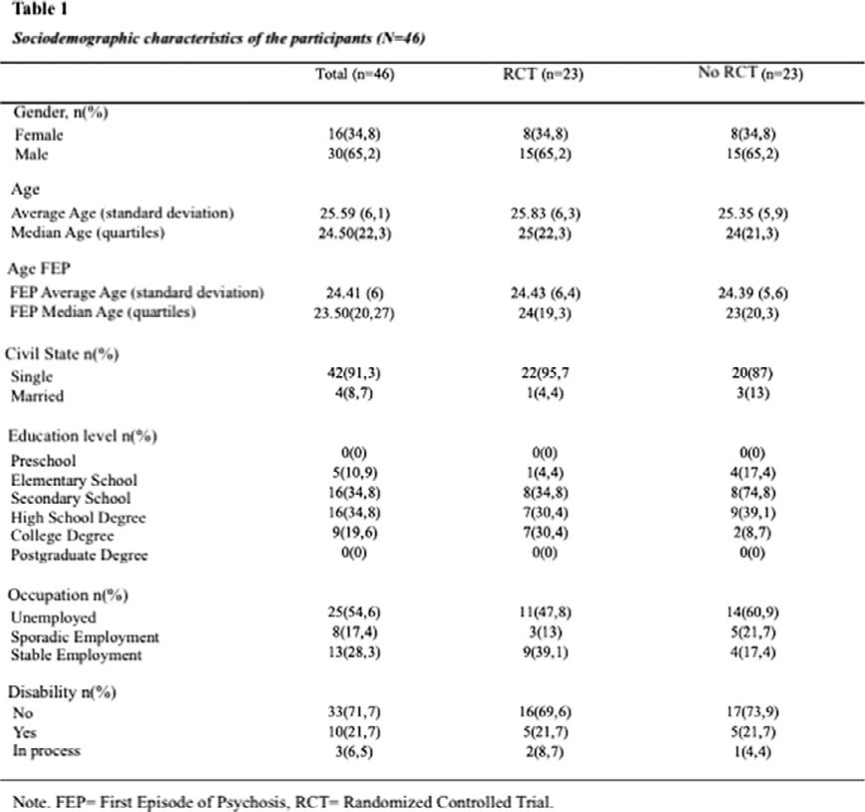

**Image 2:**

**Figure:**
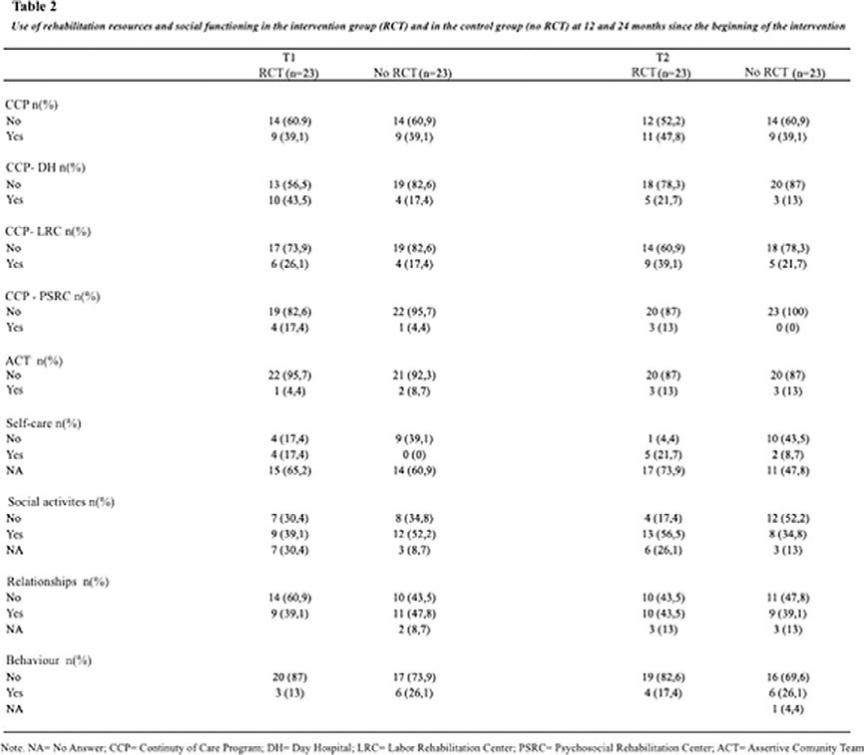

**Conclusions:**

Further studies with larger sample sizes are needed in order to determine if the participation in group therapy leads to an improvement in social functioning and use of rehabilitation resources for people who have experienced a first episode of psychosis.

**Disclosure of Interest:**

None Declared

